# Impact of Manganese and Chromate on Specific DNA Double-Strand Break Repair Pathways

**DOI:** 10.3390/ijms241210392

**Published:** 2023-06-20

**Authors:** Vivien M. M. Haberland, Simon Magin, George Iliakis, Andrea Hartwig

**Affiliations:** 1 Department of Food Chemistry and Toxicology, Institute for Applied Biosciences, Karlsruhe Institute of Technology (KIT), 76131 Karlsruhe, Germany; vivien.haberland@googlemail.com; 2 Institute of Medical Radiation Biology, Medical School, University of Duisburg-Essen, 45122 Essen, Germany; simon.magin@uk-essen.de (S.M.); georg.iliakis@uk-essen.de (G.I.)

**Keywords:** chromate, manganese, DNA DSB repair, HR, MMEJ, SSA, NHEJ, U2OS reporter-assay, BRCA1, RAD51, RAD54

## Abstract

Manganese is an essential trace element; nevertheless, on conditions of overload, it becomes toxic, with neurotoxicity being the main concern. Chromate is a well-known human carcinogen. The underlying mechanisms seem to be oxidative stress as well as direct DNA damage in the case of chromate, but also interactions with DNA repair systems in both cases. However, the impact of manganese and chromate on DNA double-strand break (DSB) repair pathways is largely unknown. In the present study, we examined the induction of DSB as well as the effect on specific DNA DSB repair mechanisms, namely homologous recombination (HR), non-homologous end joining (NHEJ), single strand annealing (SSA), and microhomology-mediated end joining (MMEJ). We applied DSB repair pathway-specific reporter cell lines, pulsed field gel electrophoresis as well as gene expression analysis, and investigated the binding of specific DNA repair proteins via immunoflourescence. While manganese did not seem to induce DNA DSB and had no impact on NHEJ and MMEJ, HR and SSA were inhibited. In the case of chromate, the induction of DSB was further supported. Regarding DSB repair, no inhibition was seen in the case of NHEJ and SSA, but HR was diminished and MMEJ was activated in a pronounced manner. The results indicate a specific inhibition of error-free HR by manganese and chromate, with a shift towards error-prone DSB repair mechanisms in both cases. These observations suggest the induction of genomic instability and may explain the microsatellite instability involved in chromate-induced carcinogenicity.

## 1. Introduction

Manganese is an essential trace element involved in many enzymatic reactions, and is required for energy metabolism, reproduction, and development. While adequate amounts are easily obtained via food, manganese overload can arise due to the inhalation or ingestion of elevated levels, due to iron deficiency or due to impaired excretion as a consequence of hepatic dysfunction [1]. The industrial use of the metal ranges from the steel production and processing to the manufacture of batteries. In addition, manganese is included as an ingredient in herbicides and fungicides such as mancozeb. The most critical effect after long-term exposure via inhalation at workplaces, for example in welders, is neurotoxicity. In so-called manganism, manganese is enriched in the basal ganglia of the brain and accumulates in the mitochondria, presumably due to the impairment of the respiratory chain in the mitochondria and the associated generation of ROS [2]. With regard to DNA damage induction and processing, manganese has been shown to induce DNA single strand breaks and to inhibit PARP-1, a central DNA damage signaling enzyme involved in basically all major DNA repair pathways [3].

Hexavalent chromium compounds occur primarily in industrial processes such as the production of cement or leather tanning as contamination and residues, with inhalation being the main route of occupational exposure. Hexavalent chromium induces lung cancer, tumors in the nasal epithelium and decreased fertility, and has been classified as carcinogenic to humans by IARC (Group 1) [4]. Due to its structural similarity to phosphates and sulfates, hexavalent chromium is readily taken up by cells via anion transporters. Subsequently, Cr(VI) is reduced to Cr(III) in three one-electron steps via Cr(V) and Cr(IV) by intracellular reductants such as ascorbic acid (Asc) or glutathione (GSH). During the reduction, reactive intermediates are formed, which may lead to DNA damage [5]. Cr(III) forms binary (Cr(III)-DNA) and ternary DNA adducts, which include Asc, GSH, cysteine, or histidine, and exert a high mutagenic potential. During replication, the ternary DNA adducts provoke DNA base mismatches, recognized by the mismatch repair protein complex, which block the replication fork and thus generate DNA double-strand breaks (DSB) [6,7,8]. Mismatch repair deficiency results in microsatellite instability, increasing the risk of tumor development [9,10,11].

Both manganese and Cr(VI) induce DNA single strand breaks (SSB), and Cr(VI) additionally leads to the generation of DNA DSB. However, not much is known about the impact of both metals on the repair of DNA DSB. DNA DSB are among the most serious types of DNA damage, as they are lethal to the cells if not repaired. On the other hand, errors can be introduced during repair, since both DNA strands are affected. In principle, DNA DSB can be repaired by HR, NHEJ, MMEJ, or SSA. While the largely error-free HR uses homologous sequences on the sister chromatid, NHEJ, MMEJ, and SSA are rather error-prone pathways [12,13,14]. Even though there are some indications that Cr(VI) may affect HR [15,16], a systematic investigation is still missing.

The aim of the present study was to elucidate the impact of manganese and chromate on the induction of DNA DSB and respective DNA repair mechanisms. We used pulsed field gel electrophoresis (PFGE) to assess the induction and repair of DSB by manganese. Furthermore, endonuclease inducible reporter cell lines were used to assess the activation or inhibition of the major DNA DSB repair mechanisms by manganese and Cr(VI). In the next step, the impact of the metal compounds on specific proteins involved in HR was investigated, namely BRCA1, RAD51 and RAD54. In the case of manganese, gene expression profiles were established additionally to elucidate the impact on oxidative stress response and DNA repair factors on the transcriptional level. Our results demonstrate an induction of DSB by Cr(VI), but not by manganese. With respect to the repair of these lesions, most strikingly, the largely error-free HR was inhibited, starting at non-cytotoxic concentrations of manganese and Cr(VI), while error-prone pathways were fully active, with MMEJ being even more activated in the case of Cr(VI).

## 2. Results

### 2.1. Manganese

#### 2.1.1. Cytotoxicity

**Figure 1:**
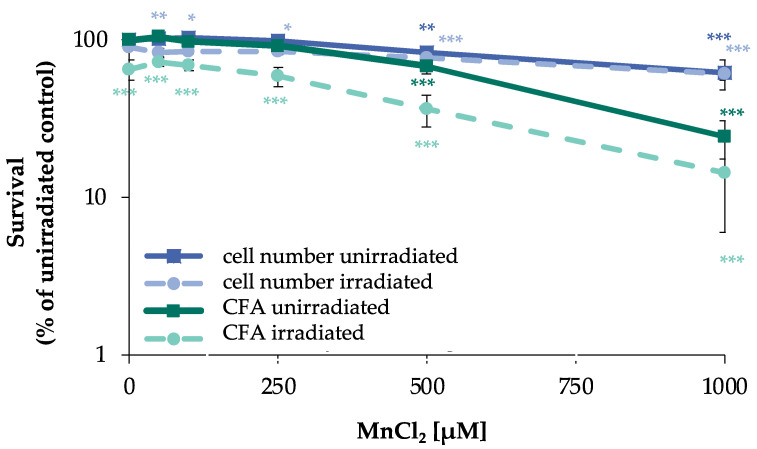
Impact of manganese on cell number and CFA in HeLa S3 cells after irradiation. Cells were pre-incubated with MnCl_2_ for 24 h, irradiated with 1 Gy and post-incubated in the presence of MnCl_2_ for 8 h. Cells were subsequently trypsinized, counted (cell number), and reseeded for assessment of colony forming ability (CFA). Results are normalized to cell number or CFA of the untreated control. Shown are mean values of three independent experiments performed each with double determination ± SD. Statistical analysis between manganese treatment and corresponding controls (* *p* ≤ 0.05, ** *p* ≤ 0.01, *** *p* ≤ 0.001) were performed by one-way ANOVA with post hoc Dunnett T.

To establish a suitable concentration range for manganese, the acute and long-term cytotoxicity on cell number and colony forming ability (CFA) were examined. To ensure uptake, cells were pre-incubated with manganese for 24 h. When assessing the impact on DSB generation and their repair, IR doses were chosen according to the sensitivity of the respective test system. Thus, 80 Gy were selected in the case of PFGE, and the impact of manganese was investigated after 24 h pre- and 8 h post-incubation. Under these conditions, no significant impact on acute toxicity as determined by cell number or ATP content after irradiation alone nor in combination with up to 500 µM manganese was observed. Since the detection of foci formation is far more sensitive, the cells were pre-incubated with manganese, irradiated with only 1 Gy to generate DNA DSB and post-incubated for 8 h in the presence of manganese. A total incubation time of 32 h with manganese was also chosen for non-irradiated cells. In addition, an incubation time of 72 h was investigated to assess the long-term toxicity. With respect to cell number, a concentration-dependent cytotoxicity of MnCl_2_ was observed, with slight toxicity starting at 500 µM. Irradiation at 1 Gy had no pronounced impact on cell number, neither alone, nor after combined treatment with MnCl_2_. Regarding CFA, toxicity also started at 500 µM MnCl_2_. However, for this endpoint, a clear reduction was observed after irradiation with 1 Gy, provoking a decrease to about 65% of untreated control. The combined treatment with manganese revealed about additive effects (Figure 1). No toxicity was observed at either endpoint after 72 h incubation with up to 250 µM MnCl_2_.

#### 2.1.2. Induction and Repair of DNA DSB

**Figure 2:**
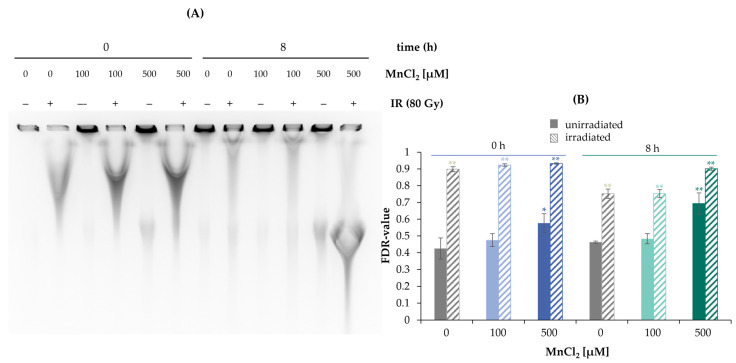
Impact of manganese on the repair of IR-induced DSB measured by PFGE. HeLa S3 cells were pre-incubated with MnCl_2_ for 24 h and irradiated with 80 Gy. Cells were harvested directly afterwards or post-incubated in the presence of MnCl_2_ for further 8 h. (**A**) DNA fragments were separated by gel electrophoresis. Shown is one representative gel from three determinations. (**B**) The fraction of DNA released (FDR) value was quantified, describing the ratio of DNA fraction migrated in the gel to the total DNA content as an indicator for DNA DSB. Shown are mean values of three independent experiments, each performed in duplicate ± SD. Statistical analysis between the respective treatments and controls (* *p* ≤ 0.05, ** *p* ≤ 0.01) were performed by one-way ANOVA with post hoc Dunnett T.

To examine the general impact of MnCl_2_ on the induction and repair of DNA DSB, PFGE was performed (Figure 2A,B). By this approach, DNA fragments migrating into the gel are indicative of DNA DSB (Figure 2A), which can be (semi)-quantified by the so-called fraction of DNA released (FDR) value, the ratio of DNA content in the gel to the total DNA content (Figure 2B). DNA DSB were generated by X-rays and analyzed either immediately (0 h) or after 8 h to follow their induction and repair, respectively. In the absence of manganese, DNA DSB were induced directly after irradiation (0 h) with 80 Gy, reaching an FDR value close to 1 and showing substantial repair after 8 h post-incubation. In non-irradiated cells, treatment with 500 µM MnCl_2_ induced a small but significant increase in FDR, suggestive of elevated levels of DSB. This effect even increased after additional 8 h incubation with manganese. In the case of pre-incubation with manganese and subsequent irradiation, no major changes were evident at 100 µM manganese directly after irradiation or after additional 8 h incubation; however, elevated fragmentation was visible at 500 µM at the second time point.

#### 2.1.3. Impact on Specific DSB Repair Pathways

**Figure 3:**
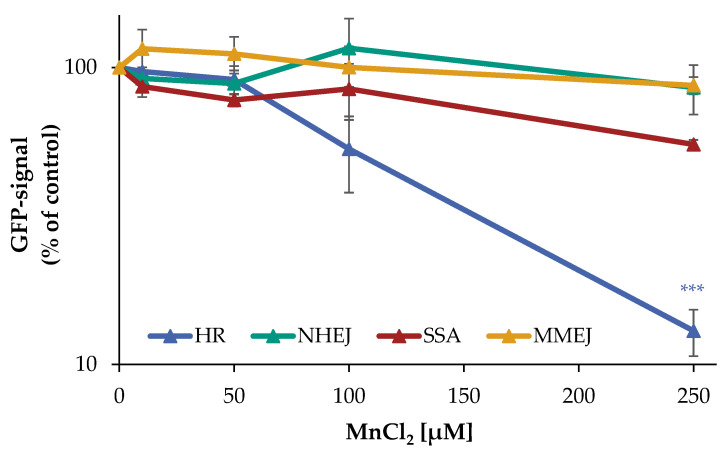
Impact of manganese on the repair of DSB measured by U2OS reporter-assay. U2OS cells were transfected with ISce-I for 6 h. After removing the transfection cocktail, the cells were incubated with MnCl_2_ for 66 h. Cells were subsequently trypsinized, harvested, and GFP-active cells were quantified via flow cytometry. The results are normalized to the transfected control. Shown are mean values of three independent experiments performed in double determination ± SD. For each treatment, 50,000 events were counted. Statistical analysis between manganese treatment and corresponding controls (*** *p* ≤ 0.001) were performed by one-way ANOVA with post hoc Dunnett T.

However, since the PFGE does not allow any differentiation between the induction of DNA DSB and their repair, as well as a potential impact on specific DNA DSB repair mechanisms, the individual repair pathways HR, NHEJ, SSA, and MMEJ were examined using an DSB repair reporter assay [17,18]. U2OS cells with specific, inactive GFP expression cassettes, interrupted by a restriction site of ISce-I, were transfected with ISce-I to induce DNA DSB. After successful repair of these DNA DSB by a specific repair pathway, expression of active GFP can be detected by flow cytometry. To obtain a maximum GFP signal, cells were incubated for 66 h after transfection. The numbers of GFP positive cells were quantified in the absence and presence of manganese. While error-prone NHEJ and MMEJ were unaffected by manganese, decreases in the number of GFP positive cells for the largely error-free HR at 100 µM to about 50% and at 250 µM manganese to about 13% of the untreated control were observed. For SSA, at 250 µM manganese a reduction to about 55% compared to the untreated control was found (Figure 3).

The results show that manganese does not affect the NHEJ and MMEJ repair pathways, whereas the HR appears to be almost completely inhibited by manganese. Moreover, at higher manganese concentrations, there is a tendency for the impairment of SSA.

#### 2.1.4. Impact on the Association of Specific DNA Repair Factors

The specific impact of manganese on mostly error-free HR was examined via immunofluorescence (IF) staining of RAD51 and RAD54, providing information on the recruitment of specific repair proteins to the sites of DNA damage. Analysis was performed after 24 h pre-incubation with manganese, 1 Gy irradiation, and 8 h post-incubation in the presence of manganese; as a second time point, 72 h (64 h pre-incubation plus 8 h post-incubation) was chosen to employ comparable conditions to the U2OS reporter cell assay. Cells in the G2 phase were evaluated exclusively, as identified by centromere protein F (CENP-F) staining via flow cytometry, since HR is restricted to the late S and G2 phases of the cell cycle [19].

**Figure 4:**
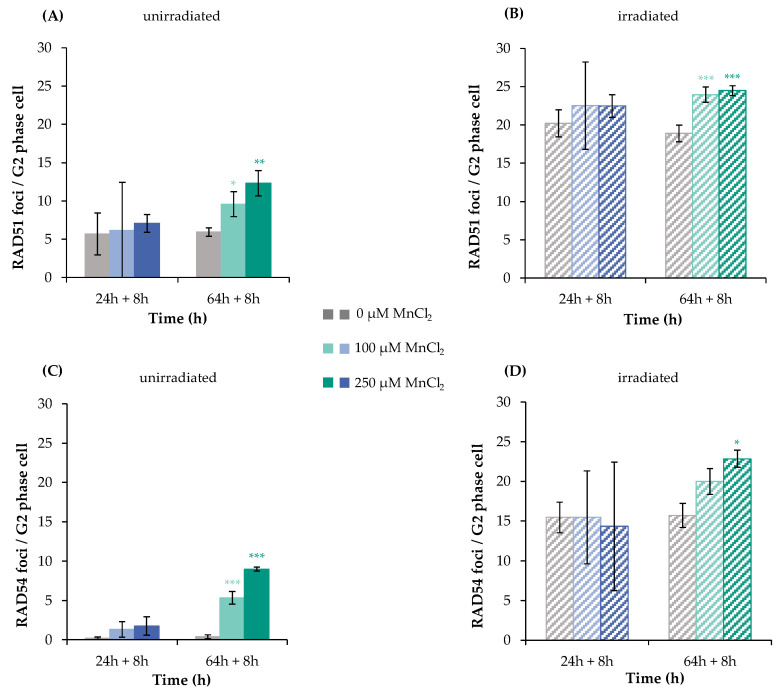
RAD51 and RAD54 foci formation after manganese treatment and irradiation with 1 Gy in HeLa S3 cells. Cells were pre-incubated with MnCl_2_ for 24 h or 64 h (**A**,**C**), irradiated with 1 Gy (**B**,**D**) and post-incubated in the presence of MnCl_2_ for 8 h. Cells were stained against RAD51 (**A**,**B**) or RAD54 (**C**,**D**). G2 cells were identified via CENP-F staining. Shown are unirradiated (**A**,**C**) and irradiated (**B**,**D**) cells. The data represent mean values of three independent experiments performed in double determination ± SD. For each time point and treatment, respective foci were counted in 40 G2 phase cells. Statistical analysis between manganese treatment and corresponding controls (* *p* ≤ 0.05, ** *p* ≤ 0.01, *** *p* ≤ 0.001) were performed by one-way ANOVA with post hoc Dunnett T. Pictures displaying foci formation are shown in Supplementary Figures S1 and S2.

In a first step, RAD51 was chosen as a marker protein, since it is involved exclusively in HR. Since it carries critical thiol groups, it may represent a sensitive target for metal ions [20,21]. Figure 4A,B as well as Figure S1 show the results of IF staining of RAD51 after incubation with MnCl_2_ (100 µM and 250 µM) with or without additional irradiation. While the unirradiated controls exhibited about 6 foci per cell, 1 Gy irradiation increased this frequency to about 20 foci at 8 h after IR for both pre-incubation schedules. Concerning manganese treatment alone, no impact was seen after 32 h incubation, but a significant increase of foci number after 72 h. After combined treatment, no impact by manganese was detected after 32 h and a slight increase after 72 h pre-incubation, resembling nearly additive effects (Figure 4A,B). These data add further evidence for the increase in DNA DSB by manganese after long-term exposure; binding of RAD51 to the sites of DNA damage was not impaired. In the next step, it was investigated whether or not the subsequent recruitment of RAD54 may be affected. RAD54 is also involved exclusively in HR; it binds to RAD51 and mediates its degradation [22]. This protein contains a zinc-binding structure in its C-terminal domain, which could also be targeted by metals ions.

As shown in Figure 4C,D, non-irradiated controls showed only about one RAD54 focus per cell. Binding of this protein was not increased after 32 h manganese treatment, but significantly elevated after 72 h. Upon irradiation, about 15 foci per cell were detected, with no impact of 32 h manganese pre-treatment, and again about additive levels in combination with 72 h manganese exposure at 100 and 250 µM. Thus, comparable to the results with RAD51, manganese does not impair RAD54 binding; furthermore, the findings suggest an accumulation of DNA DSB after 72 h treatment with manganese.

#### 2.1.5. Impact on Gene Expression Profiles

**Figure 5:**
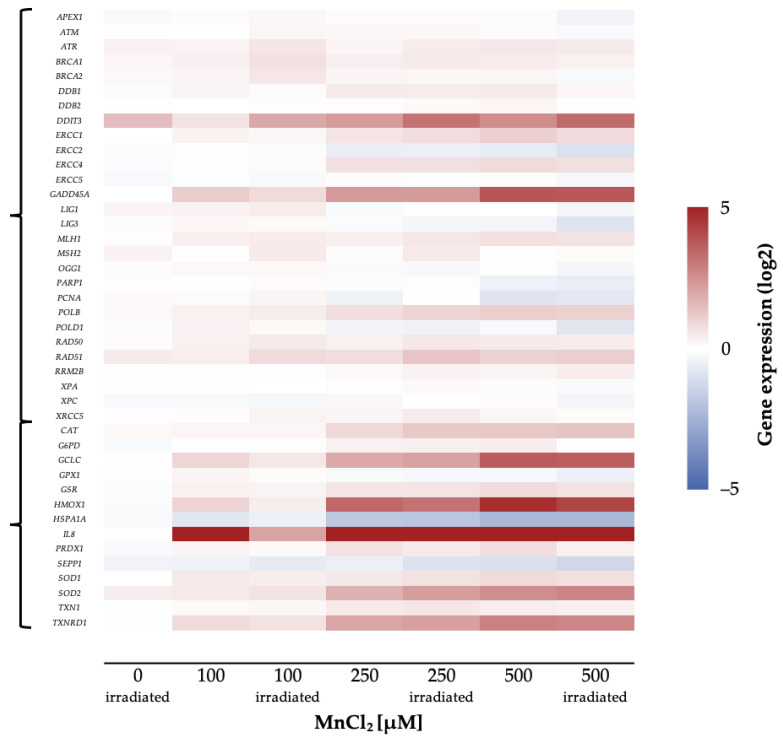
Overview of the impact of manganese on HeLa S3 cells using a high-throughput RT-qPCR. Cells were pre-incubated with MnCl_2_ for 24 h, irradiated with 1 Gy and post-incubated in the presence of MnCl_2_ for 8 h. Genes have been clustered into groups associated with DNA damage response and oxidative stress response. Displayed are the log2-fold changes of relative gene expression as a heatmap. Red represents an enhanced expression and blue represents a down-regulation of different genes. Shown are the mean values of at least three independently conducted experiments.

To investigate the impact of manganese on the transcriptional toxicity profiles related to genomic stability, a high-throughput RT-qPCR test system was applied. This method allows simultaneous analysis of 95 genes in 96 samples, comprising markers related to DNA damage response, oxidative stress response, inflammation, apoptosis, and cell cycle regulation, as well as metal homeostasis [23]. A change in the expression levels of respective genes by a factor of 2 is considered relevant. Figure 5 displays the impact of MnCl_2_ on the gene expression profile of HeLa S3 cells, visualizing genes related to the DNA damage response and oxidative stress response in a heatmap.

Most strikingly, genes related to inflammation and oxidative stress response were induced in a concentration-dependent manner. The strongest impact was observed for the inflammatory marker interleukin 8 *(IL8)*, which was induced up to about 500-fold.

In the case of genes related to oxidative stress response, the expression of *GCLC* and *HMOX1*, coding for glutamate cysteine ligase and heme oxygenase 1, respectively, were induced up to 7-fold, while the transcript level of heat shock protein A1A *(HSPA1A)* was decreased by 50%. In addition, *SOD2* and *TXNRD1*, coding for manganese-dependent superoxide dismutase 2 and thioredoxin reductase, showed an up to 2-fold induction.

Furthermore, genes coding for DNA damage response proteins (*DDIT3*, *GADD45A)* were induced by MnCl_2_ in a concentration-dependent manner. The genotoxic stress marker *DDIT3* showed a 4-fold induction, whereas *GADD45A*, as a second marker for genotoxic stress, showed a 5-fold increase of expression. In contrast, genes coding for specific DNA repair proteins were not induced, independent of the pathway (Figure 5). Other factors with dose-dependent increased expression upon manganese treatment were cell cycle regulators such as *CDKN1A* as well as pro-apoptotic genes like *BBC3* and *PMAIP1*.

### 2.2. Hexavalent Chromium

#### 2.2.1. Cytotoxicity

**Figure 6:**
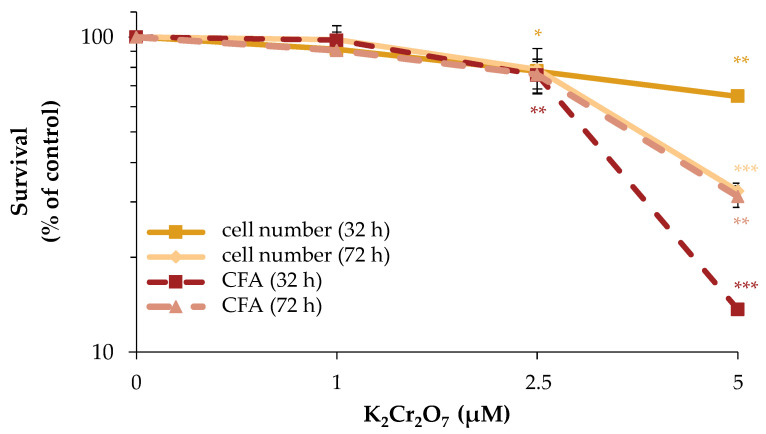
Impact of Cr(VI) on cell number and CFA of HeLa S3 cells. Cells were incubated with K_2_Cr_2_O_7_ for 32 h or 72 h. Cells were subsequently trypsinized, counted (cell number) and reseeded for assessment of colony forming ability (CFA). Results are normalized to cell number or CFA of the untreated control. Shown are mean values of three independent experiments performed in double determination ± SD. Statistical analysis between chromate treatment and corresponding controls (* *p* ≤ 0.05, ** *p* ≤ 0.01, *** *p* ≤ 0.001) were performed by one-way ANOVA with post hoc Dunnett T.

To investigate the acute and long-term cytotoxicity of Cr(VI)_,_ its impact on cell number and CFA was examined. Total incubation times of 32 h and 72 h were chosen, following the same schedule as outlined for manganese. With respect to cell number, a concentration-dependent cytotoxicity of Cr(VI) was observed, revealing a reduction to about 80% of control starting at 2.5 µM K_2_Cr_2_O_7_ after 32 h as well as after 72 h of incubation. At 5 µM, the cell number declined significantly to 65% after 32 h and to 35% after 72 h. Regarding CFA, at 2.5 µM, the number of colonies decreased to about 75% of control at both time points. At 5 µM, a strong and significant cytotoxic effect was evident, with a decrease to 15% and 30% of control after 32 or 72 h, respectively (Figure 6).

#### 2.2.2. Impact on Specific DSB Repair Pathways

**Figure 7:**
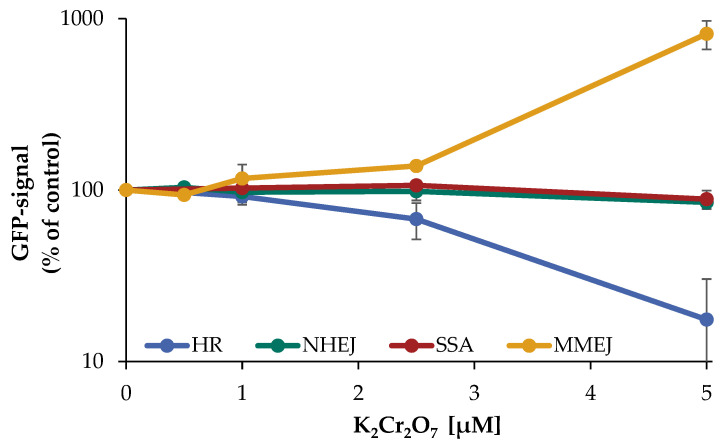
Impact of Cr(VI) on different DSB repair pathways determined by U2OS reporter-assay. U2OS cells were transfected with ISce-I for 6 h. After removing the transfection cocktail the cells were incubated with K_2_Cr_2_O_7_ for 66 h, to obtain a maximum GFP signal. Cells were subsequently trypsinized, harvested, and GFP-active cells were quantified via flow cytometry. The results are normalized to the transfected control. Shown are mean values of three independent experiments performed with double determinations ± SD. For each treatment 50,000 events were counted. Statistical analysis between chromate treatment and corresponding controls were performed by one-way ANOVA with post hoc Dunnett T.

Following the determination of a suitable concentration range, the impact of Cr(VI) on the repair of DNA DSB in the different U2OS reporter cell lines specifically detecting HR, NHEJ, SSA, or MMEJ was investigated as described above. In the case of NHEJ as well as SSA, almost no impact of Cr(VI) was observed, with 85% of control signal up to 5 µM K_2_Cr_2_O_7_. In contrast, a decrease in the GFP signal of HR was evident starting at 2.5 µM (65% of control) and dropping further to 15% at 5 µM, indicating an almost completely inhibited HR. Under the same conditions, the signal of MMEJ was elevated in a concentration-dependent manner to 140% at 2.5 µM and even 8-fold after treatment with 5 µM K_2_Cr_2_O_7_ (Figure 7).

#### 2.2.3. Impact on the Association of Specific DNA Repair Factors

To investigate the impact of Cr(VI) on the binding of specific DNA repair factors, BRCA1, RAD51, as well as RAD54 were selected as marker proteins of HR and respective foci were quantified by immunofluorescence.

HeLa S3 cells were incubated with Cr(VI) for 24 h or 64 h, irradiated with 1 Gy to induce DNA DSB, and post-incubated for 8 h to follow repair. In addition, experiments with non-irradiated, but Cr(VI)-treated cells were included, with incubation times of 32 h or 72 h to comply with the results obtained in the reporter gene assay.

**Figure 8:**
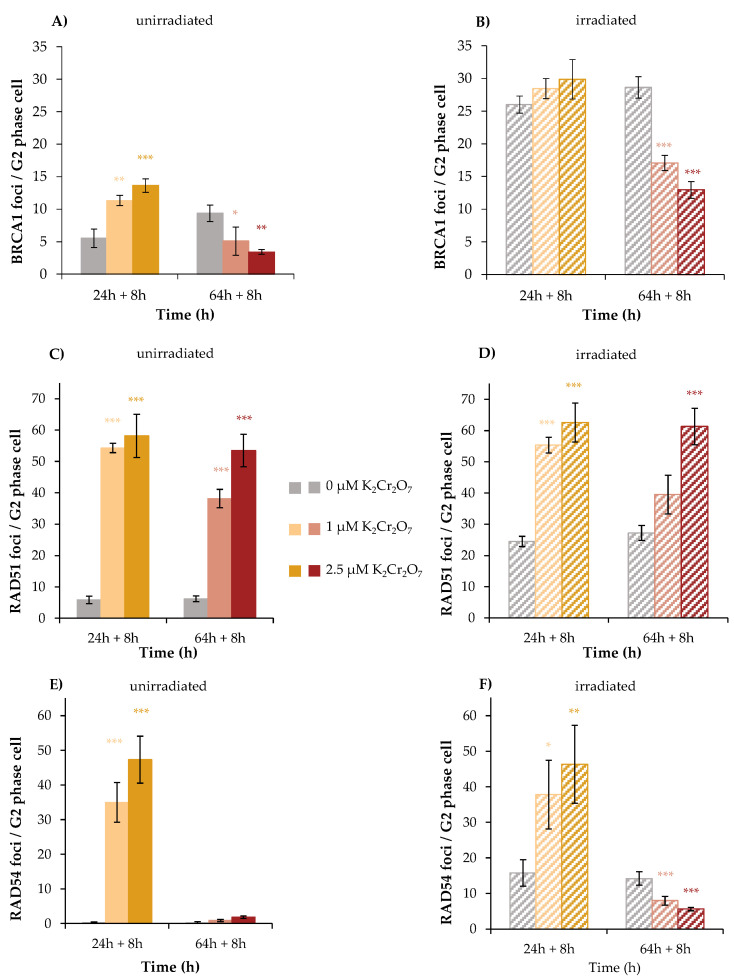
BRCA1, RAD51, and RAD54 foci formation after Cr(VI) treatment and irradiation with 1 Gy in HeLa S3 cells. Cells were pre-incubated with K_2_Cr_2_O_7_ for 24 h or 64 h (**A**,**C**,**E**), irradiated with 1 Gy (**B**,**D**,**F**), and post-incubated in the presence of K_2_Cr_2_O_7_ for 8 h. Cells were stained against BRCA1 (**A**,**B**), RAD51 (**C**,**D**), and RAD54 (**E**,**F**). G2 cells were identified via CENP-F staining. Shown are unirradiated (**A**,**C**,**E**) and irradiated (**B**,**D**,**F**) cells. The data represent mean values of three independent experiments performed in double determination ± SD. For each time point and treatment, foci were counted in 40 G2 phase cells. Statistical analysis between Cr(VI) treatment and corresponding controls (* *p* ≤ 0.05, ** *p* ≤ 0.01, *** *p* ≤ 0.001) were performed by one-way ANOVA with post hoc Dunnett T. Pictures displaying foci formation are shown in Supplementary Figures S3–S5.

The first protein investigated was BRCA1, since enhanced repair by MMEJ may be related to the inhibition of BRCA1 [24]. In HR, BRCA1 processes the DNA ends of the DNA DSB and subsequently recruits RAD51 monomers to the DNA DSB in complex with BRCA2 [25]. Within this study, approximately 5 foci were counted in the control and 11 or 14 foci after 32 h treatment with 1 µM or 2.5 µM K_2_Cr_2_O_7_, respectively. Additional irradiation induced 26 foci in the absence of K_2_Cr_2_O_7_, 28 foci at 1 µM K_2_Cr_2_O_7_ and 30 foci at 2.5 µM K_2_Cr_2_O_7_. This indicates an efficient binding of BRCA1, although with less than additive values after combined treatment. The picture changed considerably after 72 h treatment in unirradiated cells. While nine foci were counted in control cells, this number was significantly reduced upon treatment with K_2_Cr_2_O_7_ to five foci at 1 µM and three foci at 2.5 µM. With additional irradiation, this reduction was even more pronounced. Here, irradiated controls exerted 29 foci, and incubation with K_2_Cr_2_O_7_ reduced this number to 17 foci at 1 µM and to only 13 foci at 2.5 µM (Figure 8A,B).

The results indicate an induction of DNA DSB after 32 h incubation by Cr(VI) itself, but a diminished binding of BRCA1, most evident after 72 h treatment. This raised the question whether downstream proteins binding to BRCA1 were impaired as well. This was investigated next for RAD51. In this case, after 32 h without irradiation, six foci were detected in control cells; 54 foci and 58 foci were induced by 1 and 2.5 µM K_2_Cr_2_O_7_, respectively. Regarding irradiated cells, the number of RAD51 foci amounted to 25 in the irradiated control and were increased to 55 and 63 foci after combined treatment with 1 and 2.5 µM K_2_Cr_2_O_7_, respectively. After 72 h treatment, incubation with 1 µM K_2_Cr_2_O_7_ exhibited 38 and 53 foci were found at 2.5 µM. However, no further increase was observed after combined treatment, indicating far less than additive foci formation (Figure 8C,D).

Next, the binding of RAD54 was investigated. Here, no foci were detected in unirradiated control cells, neither at 32 h nor at 72 h. Nevertheless, after 32 h incubation, K_2_Cr_2_O_7_ induced considerable amounts of foci, namely 35 at 1 µM and 47 at 2.5 µM. Irradiated cells revealed 16 foci, while less than additive effects were observed after combined treatment with both concentrations of Cr(VI). Interestingly, after 72 h treatment, almost no foci were detected following Cr(VI) treatment, and the number of foci induced by irradiation also decreased significantly and dose-dependently after co-treatment with Cr(VI) at this time point. Altogether, the results further indicate an induction of DSB by Cr(VI), but binding of RAD54 was almost completely impaired after 72 h.

## 3. Discussion

The aim of the present study was to elucidate the impact of manganese and Cr(VI) on the induction and repair of DNA DSB, with special focus on their impact on specific DNA DSB repair pathways and on the accretion to chromatin of selected DNA repair proteins involved in these pathways. Additionally, gene expression profiles related to genomic stability were established in the case of manganese, while respective information was obtained in the case of Cr(VI) in a previous study [26]. The results indicate the induction of DNA DSB by Cr(VI), while manganese rather appeared to inhibit the repair of DSB. Pathway-specific DSB repair reporter cell assays revealed an inactivation of HR by both metals; in the case of Cr(VI), MMEJ was activated instead in a pronounced manner.

### 3.1. Manganese

The toxicity of manganese has been shown previously in different studies [3,27]. As underlying mechanisms increases in ROS formation and activation of associated signaling pathways have been postulated. Thus, manganese is able to interact with iron-sulfur clusters of proteins and displace iron from these structures; such proteins are found, for example, in mitochondrial respiratory chain complexes, where manganese preferentially accumulates at elevated exposure levels, leading to the accelerated formation of ROS during electron transport. Furthermore, the release of iron can also lead to Fenton reactions, resulting in an additional increase in ROS [28,29,30].

Similarly, the induction of DNA single strand breaks, presumably also via ROS, has been demonstrated before [3,31,32]. Nevertheless, up to now, there were no reports on the induction of DNA DSB by manganese. Considering the data presented within the present study, results from PFGE point towards elevated levels of DNA DSB at 500 µM manganese, which may be due to either an induction of DNA DSB or due to their accumulation as a consequence of repair inhibition, evident in the absence of irradiation as well as after 8 h repair period after irradiation. Nevertheless, this concentration of manganese is quite high, exerting cytotoxicity, and thus lower concentrations were applied for the other endpoints investigated. With respect to the latter, it seems that rather DSB repair inhibition plays a major role. Thus, with regard to the reporter gene experiments, even low, non-cytotoxic concentrations of manganese inhibited HR and SSA, while the other repair pathways were not affected. Furthermore, when investigating the binding of RAD51 or RAD54, no foci formation above the control was detected after 32 h treatment, neither by manganese alone, nor by combined treatment with irradiation. In contrast, elevated levels of foci formation were seen after 72 h treatment, which may be due to an accumulation of DNA strand breaks formed endogenously or generated as a response to irradiation. Altogether, these data support diminished repair of DSB. However, the results also suggest that the binding of the two proteins was not affected by manganese, even though both proteins carry critical SH groups which could be potential targets of toxic metal ions [20,33,34]. Considering the fact that HR was not completed as shown by the reporter cell experiments, it is thus possible that proteins that follow Rad54 in the process of HR are affected, such as polymerases [35]. The active center of DNA polymerases is mostly occupied by magnesium ions, and manganese has been shown previously to potentially replace magnesium ions, thereby increasing the error rate of polymerization [36,37,38,39,40,41,42]. One other potential mechanism could be the inhibition of PARP-1 shown previously, since poly(ADP-ribosyl)ation is also involved in DNA DSB repair [3]. PARP-1 may be particularly sensitive towards toxic metal ions, since one of the three zinc binding structures in its DNA binding domain, namely ZnF-1, appears to be not occupied with zinc under basal conditions [43,44].

The general picture of manganese toxicity, namely the involvement of ROS and the induction of DNA damage [30,45,46,47,48], is also mirrored in the gene expression profiles. Here, especially evident was the up-regulation of the inflammatory gene *IL-8* at the lowest concentration of 100 µM, of the oxidative stress response genes *HMOX1* and *GCLC* at 250 µM as well as the DNA damage signaling genes *GADD45A* and *DDIT3*, while specific DNA repair factors were not or only marginally affected, not reaching two-fold expression changes.

### 3.2. Hexavalent Chromium

The toxicity, genotoxicity, and processes leading to carcinogenicity of Cr(VI) have been investigated for many years. Important steps include the uptake via anion channels, the intracellular reduction to Cr(III), and the formation of ternary DNA adducts, including the intracellular reductant Asc or GSH. During the processing of these lesions, DNA DSB are formed, presumably via the participation of DNA mismatch repair, and finally leading to microsatellite instability [49,50,51]. The induction of DNA DSB is further supported within the present study. All three repair factors signaling DNA DSB and specifically involved in HR were recruited to the sites of DNA damage, namely BRCA1, RAD51, and RAD54, after 32 h (pre)treatment with Cr(VI), with and without additional irradiation. These observations confirm the induction of DNA DSB and at the same time exclude a direct inactivation of these proteins by Cr(VI). A different picture was obtained after 72 h pre-incubation with Cr(VI). While RAD51 foci formation was similar to the results obtained after 32 h pre-incubation with Cr(VI), the formation of BRCA1 and RAD54 foci was greatly diminished or completely inhibited, respectively. Thus, in spite of the missing binding of BRCA1 after long-term exposure, binding of RAD51 was not impaired, even though BRCA1, in complex with BRCA2, is responsible for the deposition of RAD51 [21]. Nevertheless, the foci formation of RAD51 was also less than additive after combined treatment with irradiation. Comparing our results with findings reported in the literature, upon 72 h treatment of human lung cells with zinc chromate particles, less RAD51 foci relative to the control were detected after 72 h incubation [52]. In addition, a lack of nucleoprotein filament formation was demonstrated via transmission electron microscopy [15]. Within the present study, the diminished binding of BRCA1 after long-term treatment with Cr(VI) reduced binding of RAD51, but did not prevent its binding completely; perhaps BRCA2 can provide residual association of this protein. An incomplete nucleoprotein filament formation would also explain the missing binding of RAD 54 at the 72 h time point, since the size of the filament is depending on the RAD51 protein content [53]. If the filament cannot be built up completely, RAD54 may not be able to bind to the DNA DSB complex, required for the displacement of RAD51 monomers [22]. Nevertheless, RAD51 binding may also be due to replication stress and stalled replication forks, leading to incomplete HR. One plausible explanation for the disturbed binding of BRCA1 and RAD54 after long-term treatment with Cr(VI) would consist in their down-regulation on the transcriptional level, for example, due to epigenetic alterations. In support of this theory, there is some evidence that Cr(VI) leads to hypermethylation in promotor regions of DNA repair genes and reduced expression, including *BRCA1* and *RAD51* [11,26,54]. Furthermore, hypermethylation of the *MLH1* gene has been linked to a defective mismatch repair, resulting in microsatellite instability in lung tissue of Cr(VI)-exposed workers [11]. Thus, it would be possible that RAD54 is also down-regulated via epigenetic changes, leading to a reduced expression, and thus to a lower number of foci at the sites of damage.

In addition to the induction of DNA DSB, and perhaps most important, Cr(VI) has a major impact on the repair of DNA DSB, provoking a shift in DSB repair pathways. The inhibition of HR observed in the reporter cell lines cannot be explained by diminished binding of these repair factors. Instead, similar to manganese, completion of the repair by HR appears to be inhibited and directed towards MMEJ. While it was shown previously that zinc chromate particles inhibit HR [15], within the present study, the effect of Cr(VI) on all four major pathways of DNA DSB repair has been investigated for the first time, employing respective reporter cell lines. Thus, our results add further evidence to the inhibition of HR, and in turn, demonstrate a very pronounced induction of MMEJ, while both SSA and NHEJ were not affected. The underlying reason is not clear, but could be associated with the diminished binding of BRCA1 observed after 72 h incubation, causing suppression of resection required for HR and SSA, while MMEJ can also operate with very limited resection of ends [14]; in support of this theory, it has been established previously that an impairment of BRCA1 leads to an increased induction of DSB repair by MMEJ, with the consequence of higher mutation frequencies [24]. Since HR is a largely error-free process, a shift towards the more error-prone MMEJ is likely to decrease genomic stability.

## 4. Materials and Methods

### 4.1. Materials

DMEM, penicillin/streptomycin, trypsin MnCl_2_ and K_2_Cr_2_O_7_ were obtained from Sigma-Aldrich (Steinheim, Germany) and fetal bovine serum was bought from Thermo Fisher Scientific (Dreieich, Germany). The following antibodies were used for immunostaining: Anti-BRCA1 mouse (Abcam, Cambridge, UK); Anti-CENP-F rabbit (Abcam, Cambridge, UK); Anti-CENP-F mouse (Santa Cruz, Dallas, USA); Anti-RAD51 rabbit (Abcam, Cambridge, UK); Anti-RAD54 mouse (Santa Cruz, Dallas, USA); Alexa Fluor^®^ 488-conjugated anti-mouse (Invitrogen, Darmstadt, Germany) and Cy3-conjugated anti-rabbit (Jackson Immuno Research, Newmarket, Suffolk, UK). GelRed was purchased from VWR (Darmstadt, Germany).

### 4.2. Cell Culture, Irradiation and Drug Treatment

HeLa S3 cells and U2OS cells were grown in DMEM medium containing 10% fetal bovine serum, 100 U/mL penicillin, and 100 µg/mL streptomycin at 37 °C in 5% CO_2_. Gamma-irradiation was performed with a Faxitron CellRad System (Faxitron Bioptics LLC, Tucson, AZ, USA). Logarithmic growing cells were pretreated with MnCl_2_ or K_2_Cr_2_O_7_ in cell culture medium for 24 h or 64 h, irradiated afterwards in the presence of MnCl_2_ or K_2_Cr_2_O_7_ or left unirradiated, and post-incubated for up to 8 h until fixation.

### 4.3. Cytotoxicity: Cell Number and Colony Forming Ability

Cells were trypsinized, counted, and 300 cells were reseeded in fresh medium. After 10 days, colonies were fixed with 100% ethanol (−20 °C), stained with Giemsa, and colonies containing more than 50 cells were counted and reported as percentage of control.

### 4.4. Pulsed-Field Gel Electrophoresis (PFGE)

The CHEF genomic DNA PLUG Kit (BioRad Laboratories GmbH, Munich, Germany) was applied according to the manufacturer’s instructions. Then, 5 × 10^6^ HeLa S3 cells per treatment were harvested and cell suspension buffer and melted CleanCut agarose (2%) (*v*/*v*:1/1) were added. Next, cells were embedded in agarose plugs and lysed in extraction solution buffer overnight at 50 °C. After three washing steps, plugs were transferred into the agarose gel (0.8%) containing GelRed (1:10,000). Electrophoresis was carried out applying the CHEF-DR III system (BioRad, Munich, Germany). Agarose gels were run with increasing pulse time from 5 to 5000 s over 92 h at a field strength of 1.5 V/cm at 14 °C and an angle of 120°. Visualization of DNA was performed by an imaging system (Fujifilm LAS-3000 Imager, Nishiazabu, Japan).

### 4.5. UOS Reporter-Assay

The U2OS reporter-assay includes a panel of four U2OS osteosarcoma cell lines, each for one DNA DSB repair mechanism, established originally by Gunn and Stark [18]. U2OS cells were transfected with an individual, inactive GFP expression cassette, interrupted by a restriction site for the rare-cutting endonuclease ISce-I. DNA DSB were induced by ISce-I. U2OS cells were transfected with ISce-I for 6 h with Effectene Transfection Reagent (Qiagen, Venlo, The Netherlands). Cells were additionally evaluated in parallel by cotransfecting with pEGFP-N1 to determine transfection efficiency and to serve as toxicity control. After removing transfection cocktail, cells were treated with MnCl_2_ or K_2_Cr_2_O_7_ for the duration of the experiment. Cells were harvested 72 h post transfection by trypsinization and resuspended in PBS, and the number of GFP positive cells were measured by flow cytometry (Becton Dickinson LSR Fortessa FACS, Heidelberg, Germany). In total, 50,000 events were collected.

### 4.6. Immunofluorescence

Cells grown on coverslips were fixed with 3.7% formaldehyde/PBS for 10 min at room temperature at the indicated time points. Permeabilization was carried out with 0.2% Triton X-100/PBS for 5 min at 4 °C. Subsequently, cells were treated with 2% BSA/PBS overnight at 4 °C, followed by combined incubation with anti-CENP-F antibody (1:500) and anti-BRCA1 antibody (1:1000), anti-RAD51 antibody (1:5000) or anti-RAD54 antibody (1:1000) overnight at 4 °C. All antibodies were diluted in 2% BSA/PBS. After three washing steps, cells were incubated with Alexa Fluor^®^ 488-conjugated anti-mouse antibody (1:500) and Cy3-conjugated anti-rabbit antibody (1:500) for 3 h at 37 °C, followed by three washing steps. Cells were counterstained by mounting with Vectashield Mounting Medium with DAPI (Vector Laboratories, Newark, CA, USA) and analyzed by immunofluorescence microscopy (Zeiss, Jena, Germany).

### 4.7. Gene Expression

Gene expression analyses via high-throughput RT-qPCR were performed via Fluidigm dynamic arrays on the BioMark system as described previously [23]. Data were processed using Fluidigm Real-Time PCR Analysis as well as GenEx software, Version 5.3.6. For normalization, five reference genes (*ACT*, *B2M*, *GAPDH*, *GUSB*, and *HPRT1*) were used. Expression changes of the respective genes were displayed as log2-fold change compared to untreated controls by calculating relative quantities corresponding to the ΔΔCq method [55].

### 4.8. Statistical Analysis

All results are presented as mean ± SD. Data were analyzed by one-way ANOVA with post hoc Dunnett T. Values of *p* < 0.05 were considered statistically significant.

## 5. Conclusions

In summary, the results of the present study add further insight into the direct and indirect genotoxicity of manganese and Cr(VI), with special emphasis on the induction and repair of DNA DSB. In the case of manganese, the gene expression profiles support the general picture of manganese toxicity, namely the induction of DNA damage, as well as oxidative and inflammatory stress response within the cells. The main findings concerning the impact of both metals on the induction and repair of DNA DSB are summarized in Table 1.

In the case of manganese, in spite of the induction of DNA SSB demonstrated in several studies, the induction of DSB does not appear to play a major role, most evident by the missing foci induction of the DNA repair factors RAD51 and RAD54 after 32 h incubation. In contrast, an impact on DNA DSB repair was clearly demonstrated by increased levels of respective foci after long-term treatment, indicative of accumulating DNA DSB, and supported by the results derived from PFGE. Perhaps most important, as shown for the first time by reporter cell assays, manganese inhibits the largely error-free HR, which may lead to a shift from a largely error-free HR to more error prone mechanisms of DSB repair. Regarding Cr(VI), a different picture can be drawn when compared to manganese. Here, the findings within the present study support the induction of DNA DSB, indicated by the binding of the BRCA1, RAD51, and RAD54. Nevertheless, even though all three DNA repair proteins are involved in HR, the completion of HR is inhibited as evident from the reporter gene analysis. Instead, MMEJ is activated, provoking again a shift from largely error-free HR to a rather error-prone repair pathway. The involvement of erroneous DSB repair has been clearly linked to microsatellite instability and elevated cancer risk, especially also with respect to a shift from HR to MMEJ and the involvement of inhibited mismatch repair [13,56]. Therefore, the results presented within this study may be of great importance to explain the microstallite instability involved in Cr(VI)-induced carcinogenicity.

## Figures and Tables

**Table 1:** Summary: Impact of Mn and Cr(VI) on the induction and repair of DNA DSB.

Manganese	Chromium(VI)
No induction of DNA DSB ○ Missing (additional) foci formation after 32 h treatment (RAD51 and RAD54)	Induction of DSB ○ Pronounced (additional) foci formation after 32 h treatment (BRCA1, RAD51 and RAD54)
Inhibition of DNA DSB repair ○ Increased (additional) foci formation after 72 h treatment (RAD 51 and RAD54) ○ Accumulation of DNA DSB in PFGE after irradiation	Inhibition of DNA DSB repair ○ Diminished BRCA1 binding after 72 h treatment ○ Persistent RAD51 binding after 72 h treatment; less than additive in combination with IR ○ Diminished RAD54 binding after 72 h treatment
Impact on specific DNA DSB repair pathways ○ Pronounced inhibition of HR, slight inhibition of SSA, no impact on NHEJ or MMEJ	Impact on specific DNA DSB repair pathways ○ Pronounced inhibition of HR, pronounced activation of MMEJ, no impact on SSA or NHEJ

## Data Availability

The data presented in this study are available on request from the corresponding author (A.H.) for researchers of academic institutes who meet the criteria for access to the confidential data.

## Supplementary Materials

The supporting information can be downloaded at: https://www.mdpi.com/article/10.3390/ijms241210392/s1.
