# Supplementary material for: Impact of Manganese and Chromate on Specific DNA Double-Strand Break Repair Pathways
Source: Int J Mol Sci. 2023 Jun 20;24(12):10392. doi: 10.3390/ijms241210392 (PMC10298927; doi:10.3390/ijms241210392)
Supplement: Supplementary file 1 [file ijms-24-10392-s001.zip › ijms-2391631-supplementary.pdf]

Supplementary Material

Impact of manganese and chromate on specific DNA double strand break repair pathways

Vivien M. M. Haberland [1], Simon Magin [2], George Iliakis [2] and Andrea Hartwig [1,*]

[1] Department of Food Chemistry and Toxicology, Institute for Applied Biosciences, Karlsruhe Institute of Technology (KIT), Karlsruhe, Germany; andrea.hartwig@kit.edu; vivien.haberland@googlemail.com

[2] Institute of Medical Radiation Biology, University of Duisburg-Essen, Medical School, Essen, Germany; georg.iliakis@uk-essen.de; simon.magin@uk-essen.de

* Correspondence: andrea.hartwig@kit.edu; Tel.: +49-721-608-47645

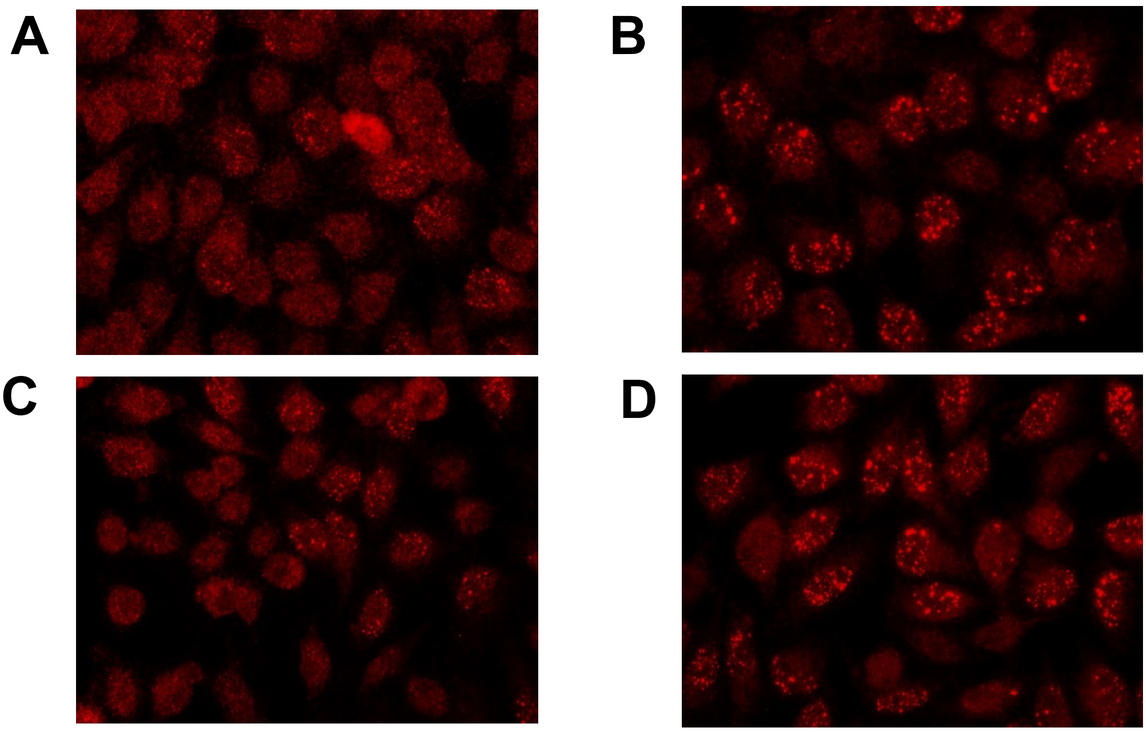

Figure S1. Impact on Rad51 foci formation by manganese and irradiation in HeLa S3 cells. A: Control; B: Irradiation with 1 Gy; C: 64 + 8 h manganese treatment (250 μM); D: 64 h pre-incubation with 250 μM manganese, 1 Gy irradiation and 8 h post-incubation with manganese.

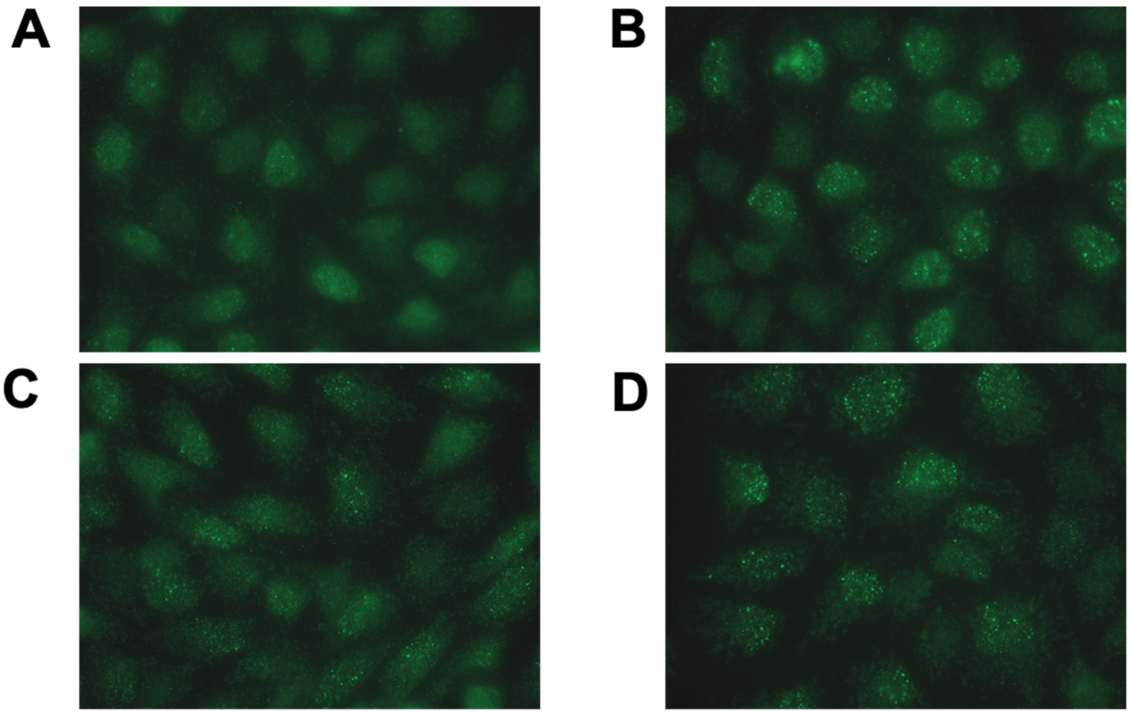

Figure S2. Impact on Rad54 foci formation by manganese and irradiation in HeLa S3 cells. A: Control; B: Irradiation with 1 Gy; C: 64 + 8 h manganese treatment (250 µM); D: 64 h pre-incubation with 250 µM manganese, 1 Gy irradiation and 8 h post-incubation with manganese.

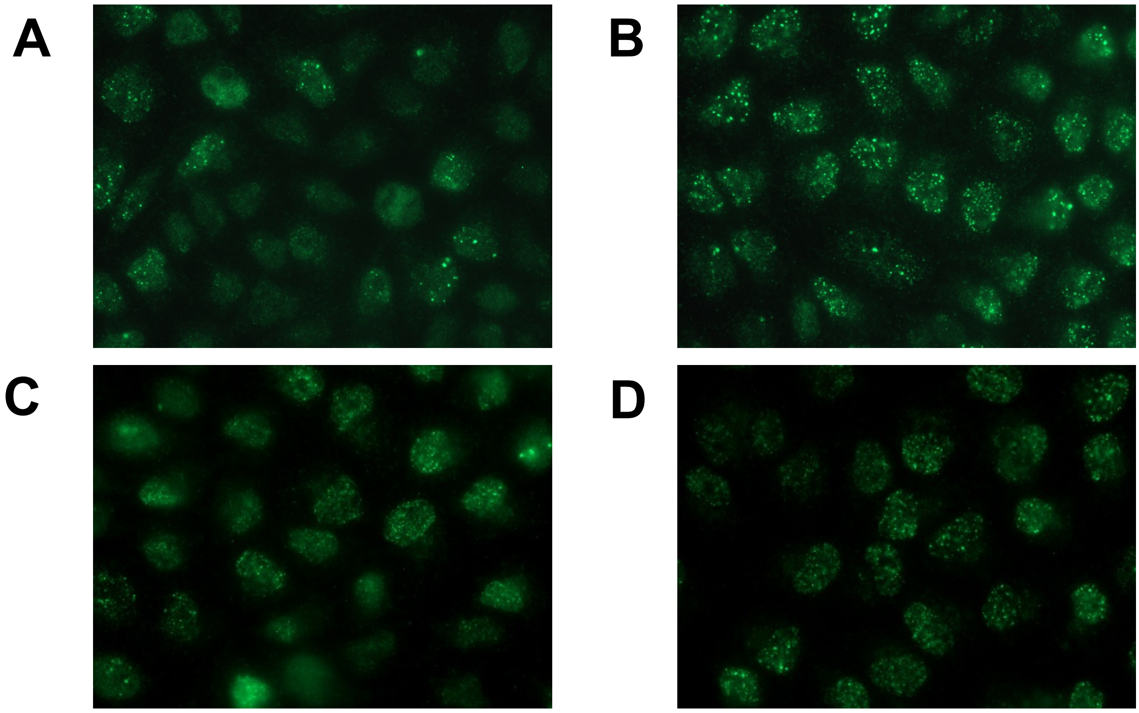

Figure S3. Impact on BRCA1 foci formation by Cr(VI) and irradiation in HeLa S3 cells. A: Control; B: Irradiation with 1 Gy; C: 64 + 8 h Cr(VI) treatment (2.5 µM); D: 64 h pre-incubation with 2.5 µM $K_2Cr_2O_7$, 1 Gy irradiation and 8 h post-incubation with $K_2Cr_2O_7$.

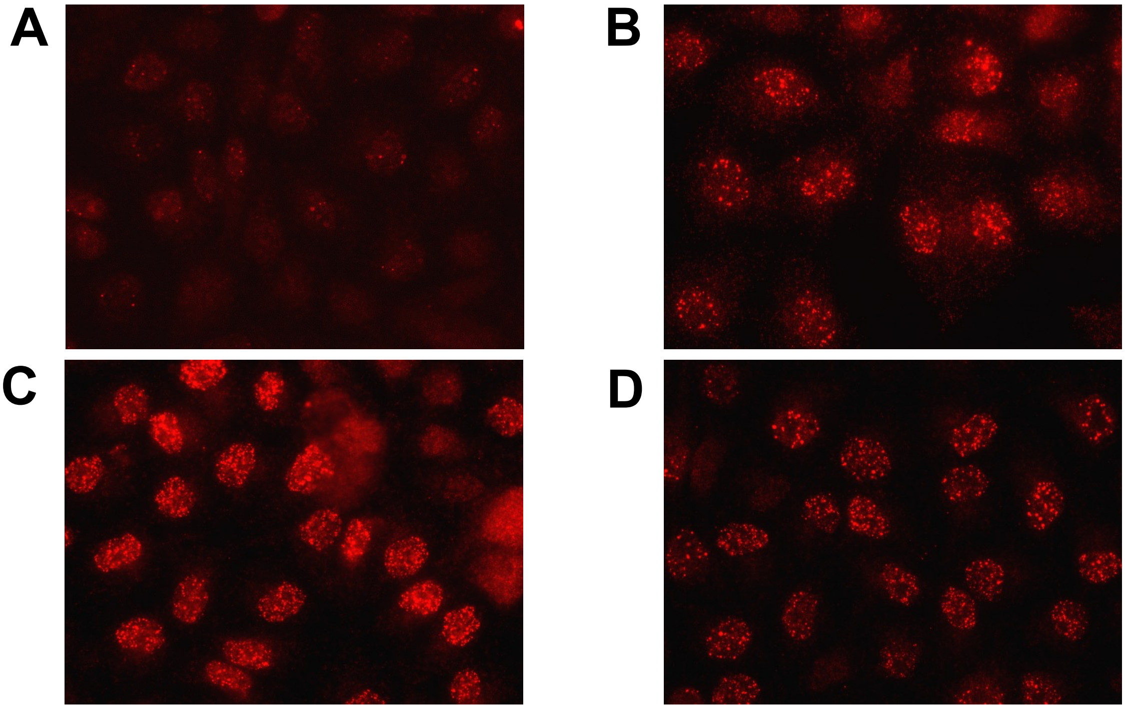

Figure S4. Impact on RAD51 foci formation by Cr(VI) and irradiation in HeLa S3 cells. A: Control; B: Irradiation with 1 Gy; C: 64 + 8 h Cr(VI) treatment (2.5 µM); D: 64 h pre-incubation with 2.5 µM $K_2Cr_2O_7$, 1 Gy irradiation and 8 h post-incubation with $K_2Cr_2O_7$.

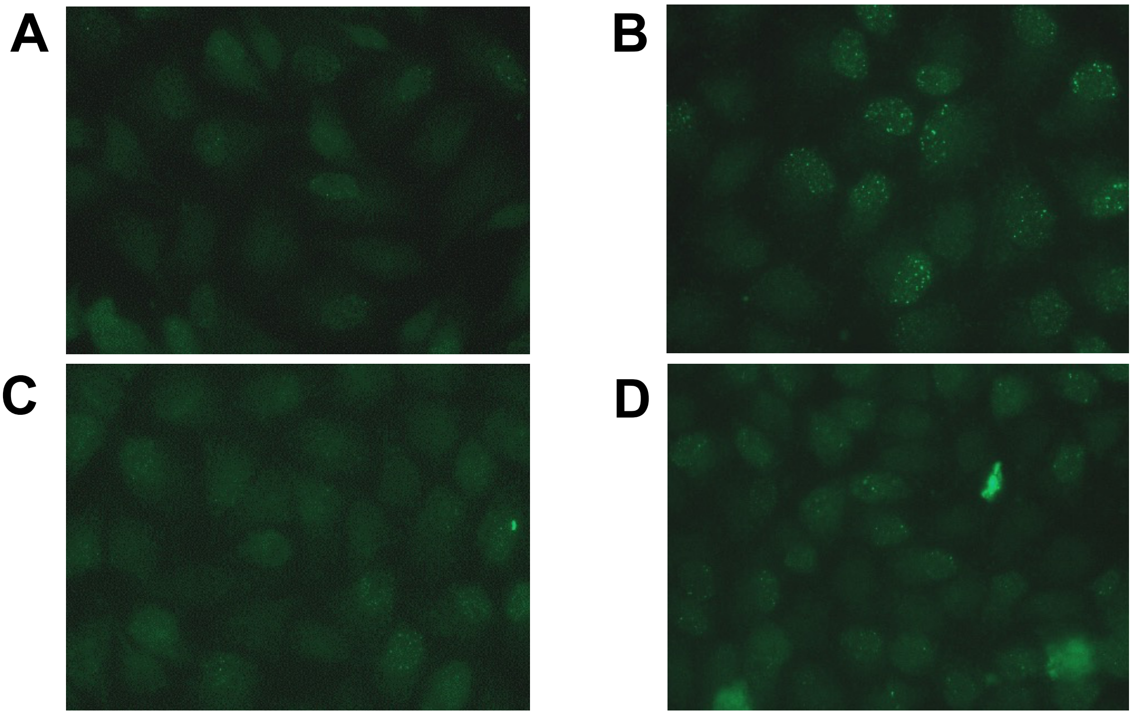

Figure S5. Impact on RAD54 foci formation by Cr(VI) and irradiation in HeLa S3 cells. A: Control; B: Irradiation with 1 Gy; C: 64 + 8 h Cr(VI) treatment (2.5 µM); D: 64 h pre-incubation with 2.5 µM $K_2Cr_2O_7$, 1 Gy irradiation and 8 h post-incubation with $K_2Cr_2O_7$.
